# Volumetric Changes at Pontic Sites After Connective Tissue Grafting: A Systematic Review

**DOI:** 10.3390/jcm15103842

**Published:** 2026-05-16

**Authors:** Jasmina Todorovic, Tim Joda, Tan Fırat Eyüboğlu, Patrícia Pauletto, Edwin Ruales-Carrera, Mutlu Özcan

**Affiliations:** 1 Clinic of Masticatory Disorders and Dental Biomaterials, Center for Dental Medicine, University of Zurich, 8032 Zurich, Switzerland; 2 Clinic of Reconstructive Dentistry, Center for Dental Medicine, University of Zurich, 8032 Zurich, Switzerland; 3 Department of Endodontics, Faculty of Dentistry, Istanbul Medipol University, 34083 Istanbul, Türkiye; 4 Faculty of Dentistry, Universidad de Las Américas, Quito 170517, Ecuador

**Keywords:** connective tissue graft, pontic site development, soft tissue augmentation, fixed dental prosthesis, volumetric analysis, systematic review

## Abstract

**Background/Objectives**: Connective tissue grafting (CTG) has been proposed to enhance ridge contour at pontic sites; however, its long-term volumetric stability remains unclear. This systematic review evaluated three-dimensional (3D) volumetric and linear soft-tissue changes over time following CTG at pontic sites. **Methods**: A systematic search was conducted in PubMed/MEDLINE, Embase, Cochrane Library, LILACS, Scopus, Web of Science, and Google Scholar. Clinical studies assessing linear and/or volumetric soft-tissue outcomes at pontic sites treated with CTG, with a minimum follow-up of six months, were included. Study selection, data extraction, and risk-of-bias assessment were performed independently by two reviewers using RoB 2 and ROBINS-I tools. Results were synthesized descriptively. **Results**: Seven studies (two randomized and five non-randomized) were included. CTG was associated with consistent early improvements in ridge contour and soft-tissue volume. Over time, minor dimensional reductions were observed, and differences between grafted and nongrafted sites tended to decrease after the initial remodeling phase. Long-term follow-up (up to 15 years) suggested overall stability of both volumetric and linear outcomes. However, the evidence is limited by a small number of studies, methodological heterogeneity, and predominance of non-randomized designs. **Conclusions**: CTG provides early soft-tissue augmentation at pontic sites, but its long-term advantage over non-grafted healing appears limited. Given the very low certainty of evidence, these findings of such studies should be interpreted with caution in clinical decision-making.

## 1. Introduction

Tooth loss caused by caries, periodontal disease, trauma, impaction, or overcrowding results in rapid and predictable remodeling of the alveolar ridge, especially within the first months after extraction. This often leads to significant horizontal and vertical dimensional loss [1,2]. These changes affect oral function, chewing efficiency, and esthetics, and can also cause tooth migration, occlusal problems, and difficulties with oral hygiene [3]. Residual ridge shape varies widely, as described in classifications by Cawood and Howell and Seibert [4,5]. This variation reflects the broad range of alveolar resorption [6,7] and influences prosthetic treatment planning.

Fixed dental prostheses (FDPs) are a common and reliable treatment option with high long-term survival rates [8,9]. In FDP therapy, the emergence profile of the pontic and its contact with the underlying ridge are crucial for esthetic balance and peri-pontic health. Various pontic designs have been described [10]; among them, the ovate pontic provides the most natural esthetic results due to its close adaptation to the ridge. However, attaining an ideal ovate form usually requires sufficient soft-tissue volume, which is often decreased in resorbed pontic sites [3,8].

Soft-tissue deficiency in the pontic area can cause mucosal collapse, food impaction, plaque buildup, inflammation, and esthetic issues [11], leading to the need for pre-prosthetic augmentation. Connective tissue grafting (CTG) is a well-established method for increasing mucosal volume. CTGs, typically taken from the palate, can reliably thicken and shape soft tissue while enabling prosthetic adjustments to improve the final ridge form [12]. The success of CTG relies on donor site selection, graft stabilization, and surgical skill [13]. Despite its widespread use, the evidence regarding the long-term volumetric stability of connective tissue grafts (CTG) at pontic sites remains inconsistent. This variability appears to stem from heterogeneity across studies, including differences in defect characteristics, surgical techniques, follow-up durations, and measurement protocols [13,14,15,16].

Furthermore, the current literature reflects an ongoing debate about the predictability of long-term soft-tissue volume stability following CTG, with studies reporting heterogeneous clinical outcomes. Such discrepancies may be attributed not only to variations in defect morphology and surgical approaches but also to differences in assessment methods, particularly between digital and conventional techniques, which can influence the accuracy and comparability of volumetric measurements.

In light of these inconsistencies and the absence of a clear consensus, a focused synthesis of the available clinical evidence is warranted. Therefore, this systematic review aims to evaluate three-dimensional volumetric and linear soft-tissue changes following CTG at pontic sites.

## 2. Materials and Methods

### 2.1. Study Design

This systematic review was conducted and is reported in accordance with the PRISMA checklist (see Supplementary Material S1) [17]. A protocol was established prior to data collection, defining the eligibility criteria, search strategies, data extraction and management methods, as well as the analytical procedures. The protocol for this review is registered in the International Prospective Register of Systematic Reviews (PROSPERO) under the number CRD420251251790.

### 2.2. Focused Question

The focused research question was: “What are the volumetric and linear soft-tissue changes over time at pontic sites following CTG?”

**Figure 1:**
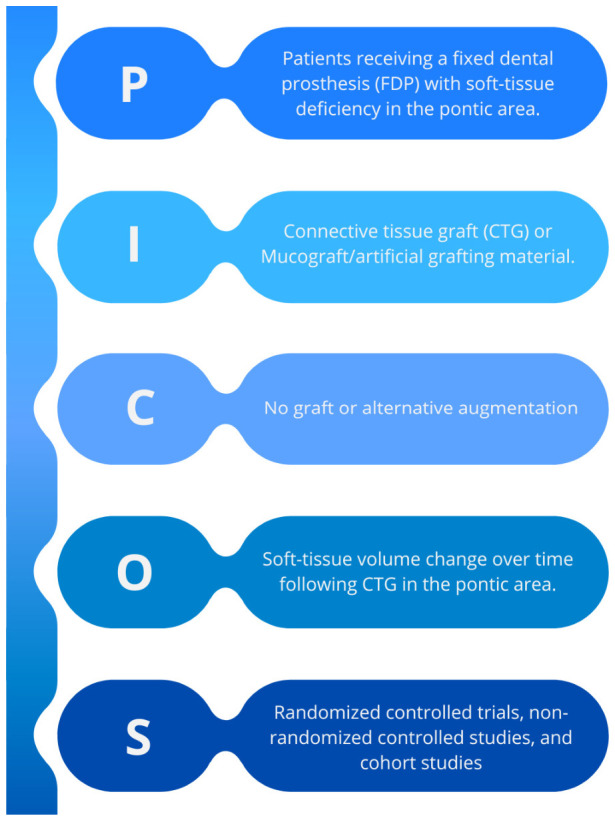
“PICOS” mnemonic as a guide to identifying the research question. Figure generated canva.com (accessed on 20 April 2026).

The question was formulated according to the PICO framework: population (patients with soft-tissue deficiency in the pontic area), intervention (soft-tissue augmentation using autogenous CTG, comparison (nongrafted sites, natural healing, or alternative approaches when reported in the included studies), and outcome (changes in linear and volumetric soft-tissue dimensions) (Figure 1).

### 2.3. Eligibility Criteria

Studies were included if they fulfilled all of the following: (1) human clinical studies; (2) evaluation of soft-tissue augmentation at pontic sites using autogenous CTG; (3) reporting of linear and/or volumetric soft-tissue outcomes; (4) minimum follow-up duration of six months; (5) minimum sample size of 10 patients; and (6) publication in English from 1990 onwards. Studies were excluded if they were case reports, animal studies, in vitro or ex vivo investigations, or if CTG was performed for indications unrelated to pontic site development (e.g., gingival recession, peri-implant soft tissue augmentation). Studies primarily focused on alternative grafting materials or ridge preservation procedures without a CTG group were also excluded. Full-text screening was performed meticulously to ensure compliance with all criteria.

### 2.4. Information Sources and Search Strategy

A comprehensive and systematic search was conducted across PubMed/MEDLINE, Embase, LILACS, Scopus, and Web of Science databases. In addition, gray literature was searched using Google Scholar. The search strategy combined controlled vocabulary terms (e.g., MeSH and Emtree, when applicable) with free-text keywords related to connective tissue grafting, pontic sites, fixed dental prostheses, volumetric assessment and soft-tissue augmentation. Additionally, backward snowballing was conducted through screening of the reference lists of all included studies and relevant reviews, and forward citation tracking was performed to identify potentially eligible studies not retrieved through electronic searches. The final electronic search was conducted in November 2022, and an update was performed in February 2026 to ensure the inclusion of recently published evidence. The full electronic search strategies for all databases are detailed in Supplementary Material S2 to ensure transparency and reproducibility.

### 2.5. Selection Process

A study selection process was conducted in two sequential stages. Initially, two reviewers (J.T. and T.J.) independently screened titles and abstracts of records identified through database searches, with the support of online screening software (Rayyan, Doha, Qatar Computing Research Institute). Subsequently, the same reviewers assessed the full texts of the selected articles to determine eligibility according to the predefined criteria. Any discrepancies between the reviewers were resolved through consultation with a third reviewer (E.R.C).

### 2.6. Data Collection Process and Data Items

Two independent reviewers (J.T. and T.J.) extracted data using a standardized template that captured study design, sample characteristics, follow-up duration, test and control group allocation, and reported linear/volumetric outcomes. Disagreements were resolved through discussion. The final dataset was synthesized qualitatively, and when possible, quantitative visualization was performed using forest plots.

### 2.7. Study Risk of Bias Assessment

Risk of bias was assessed using the Cochrane Risk of Bias tool for randomized trials (RoB 2.0) [18] and the Risk Of Bias In Non-randomized Studies of Interventions (ROBINS-I) tool [19] for the non-randomized studies. Two reviewers (J.T. and T.J.) independently evaluated each included study across the domains specified by each tool. For RoB 2.0, the following domains were assessed: bias arising from the randomization process, bias due to deviations from intended interventions, bias due to missing outcome data, bias in the measurement of the outcome, and bias in the selection of the reported result. For ROBINS-I, the domains included bias due to confounding, selection of participants, classification of interventions, deviations from intended interventions, missing data, measurement of outcomes, and selection of the reported result. Prior to the formal assessment, the reviewers discussed the application criteria for each domain and established predefined evaluation parameters to ensure consistency in judgments. Subsequently, a calibration exercise was conducted in which a subset of studies was jointly assessed to harmonize the interpretation of the signaling questions and judgment criteria. After the calibration process, the remaining studies were assessed independently. Any disagreements between the reviewers were resolved through discussion, and when consensus could not be reached, a third reviewer (P.P.) was consulted to make the final decision.

### 2.8. Outcome Definitions and Effect Measures

Given that measurement protocols can influence volumetric outcomes, the definitions of outcome measures were specified a priori and extracted consistently across the included studies.

Linear vertical outcomes included measurements related to the pontic site and adjacent abutment structures when reported. These commonly comprised pontic height (PH) as well as the vertical dimensions of the mesial (MA) and distal abutments (DA), typically obtained from cross-sectional or longitudinal slices derived from three-dimensional STL model reconstructions.

Horizontal ridge width measurements were recorded when available and were generally assessed at standardized distances apical to the mucosal margin (e.g., 1 mm, 3 mm, and 5 mm). These measurements were used to evaluate dimensional changes in the ridge contour over time.

Volumetric outcomes included mean surface distance (MD) and volumetric change (VC), which were typically calculated by superimposing serial digital models and defining a region of interest (ROI) encompassing the pontic site. The ROI-based approach allowed quantification of soft tissue contour alterations between different time points.

Across the included studies, volumetric analyses were performed using digital surface superimposition techniques applied to stereolithographic (STL) files. The acquisition of these models varied between studies, including direct intraoral digital scans or conventional impressions subsequently digitized through laboratory scanning.

### 2.9. Synthesis Methods

Due to the substantial clinical and methodological heterogeneity across the included studies, particularly regarding surgical techniques, follow-up periods, and outcome measurement methods, a quantitative synthesis (meta-analysis) was deemed inappropriate. Consequently, analyses typically required under PRISMA for meta-analyses, such as sensitivity analyses and formal exploration of statistical heterogeneity (e.g., subgroup analyses or meta-regression), were not conducted and were classified as not applicable. This decision reflects a methodological choice rather than incomplete reporting. Instead, a structured narrative synthesis was undertaken to explore patterns, variability, and gaps in the evidence, in line with PRISMA 2020 [17] recommendations for reviews where quantitative synthesis is not feasible.

The results of individual studies were tabulated and summarized in structured tables. Table 1 presents the characteristics of the included studies, including study design, sample size, follow-up duration, intervention details, and outcome measures. Table 2 summarizes the main linear, volumetric, and profilometric outcomes across studies. Visual representation of the results was performed using structured tables and graphical tools, including the PRISMA flow diagram for study selection and traffic-light plots generated with robvis for risk of bias assessment. For the synthesis, studies were grouped according to the type of outcome (linear, volumetric, and profilometric measures) and follow-up duration. This approach allowed for a structured comparison of soft-tissue changes over time across different studies.

### 2.10. Reporting Bias Assessment

The risk of bias due to missing results (reporting bias) was assessed qualitatively at the study level. We evaluated whether all prespecified outcomes described in the Methods Sections of the included studies were fully reported in the results, and whether selective outcome reporting was evident. Study protocols or trial registrations were consulted when available. In addition, discrepancies between reported outcomes, time points, and analyses were examined. The literature was comprehensively assessed through extensive searches across multiple databases, complemented by manual searches, to minimize the risk of omitting relevant studies. Given the limited number of included studies and the absence of quantitative synthesis, formal statistical methods to assess publication bias (e.g., funnel plots or regression-based tests) were not applied, as these are not recommended under such conditions.

### 2.11. Certainty Assessment

The overall certainty of the evidence was assessed using the GRADE (Grading of Recommendations Assessment, Development and Evaluation) approach [21]. The evaluation considered the following domains: risk of bias, inconsistency, indirectness, imprecision, and publication bias. Certainty judgments were performed by outcome group. Summary of Findings tables were generated using the GRADE online software (GRADEpro GDT, Version 2026).

## 3. Results

### 3.1. Study Selection

**Figure 2:**
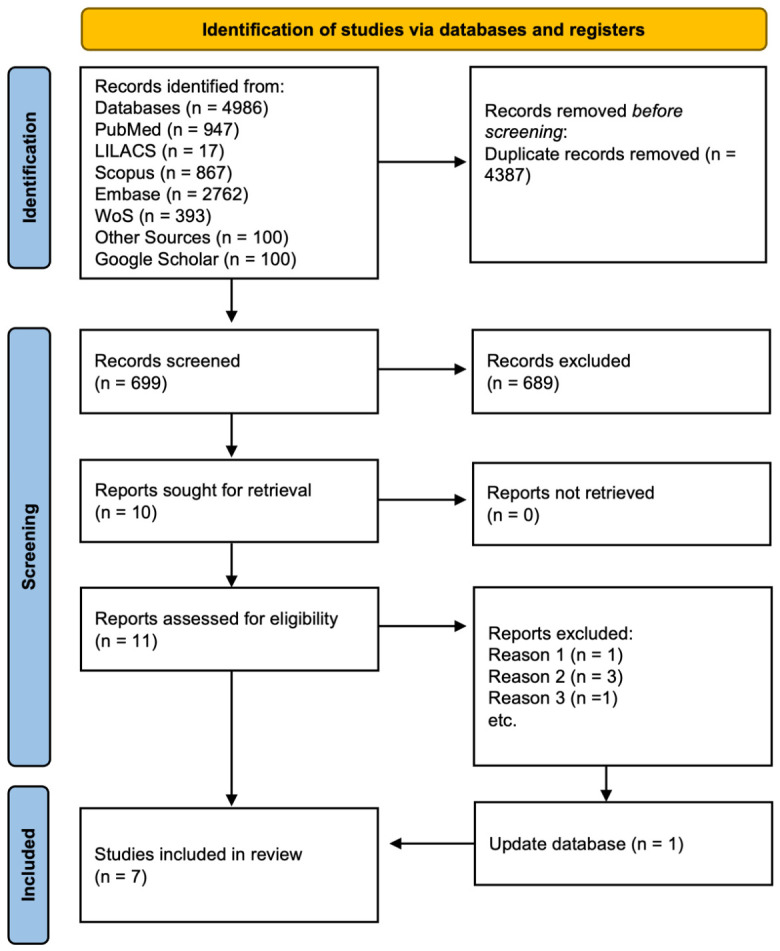
PRISMA 2020 flow diagram for new systematic reviews, which included searches of databases and registers [3,12,13,14,15,16,17,20]. This work is licensed under CC BY 4.0. To view a copy of this license, visit https://creativecommons.org/licenses/by/4.0/ (accessed on 20 April 2026).

The systematic search conducted in November 2022 identified 5086 records across the selected databases. After removing 4387 duplicate records, 699 titles and abstracts were screened. Of these, 688 records were excluded during the initial screening phase. A total of 11 full-text articles were assessed for eligibility, and six were excluded after applying the predefined inclusion and exclusion criteria (Supplementary Material S3). Consequently, 6 studies were initially included in the qualitative synthesis. An updated literature search conducted in February 2026 identified one additional eligible study, resulting in a total of seven studies included in the final qualitative analysis (Figure 2).

### 3.2. Study Characteristics

The characteristics of the included studies are summarized in Table 1. Among the included studies, two were randomized clinical trials [14,20], one was a retrospective study [16], one was a cohort study [3], and three were controlled clinical studies [12,13,15]. These controlled clinical study publications [12,13,15] were derived from the same clinical sample but reported outcomes at different follow-up periods. The seven selected studies were published between 2014 and 2025 and comprised the following reports: Schneider et al. (2014) [20], Akcalı et al. (2015) [14], Sanz-Martín et al. (2016) [13], Bienz et al. (2017) [15], Naenni et al. (2020) [16], Strauss et al. (2022) [3], and Bienz et al. (2025) [12]. For transparency, all reports are presented in Table 1.

Most studies were conducted at the University of Zurich [3,12,13,15,16,20], while one was performed at Ege University, Turkey, with volumetric analyses subsequently completed in Zurich [14]. All studies reported obtaining ethics approval and informed consent. Digital volumetric assessments used STL surface models generated either through direct intraoral scanning or digitized traditional impressions, then analyzed with Swissmeda/SMOP software.

### 3.3. Risk of Bias in Studies

**Figure 3:**
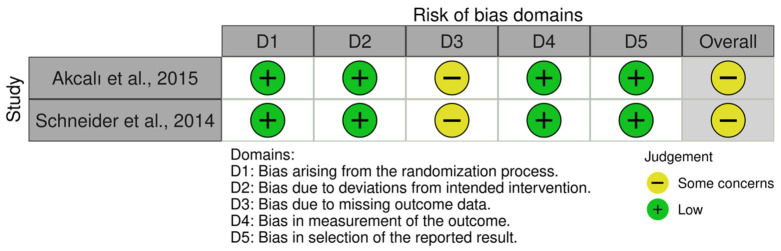
Risk of bias assessment of the randomized controlled trials using the RoB 2 tool, presented as a traffic-light plot generated with the *robvis* vizualization tool [14,20].

The risk of bias assessment for the randomized clinical trials using the Cochrane RoB 2 tool is summarized in Figure 3. Both randomized trials were judged as presenting some concerns about the overall risk of bias. The domains related to missing outcome data were judged as having some concerns in both studies.

**Figure 4:**
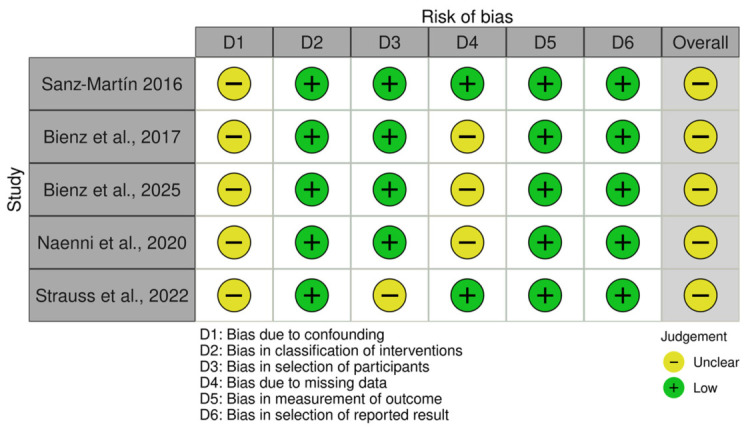
Risk of bias assessment of the non-randomized studies using the ROBINS-I tool, presented as a traffic-light plot generated with the *robvis* visualization tool [3,12,13,15,16].

The risk of bias assessment for the non-randomized studies using the ROBINS-I tool is presented in Figure 4. Overall, all included non-randomized studies were judged as presenting a moderate risk of bias. The main source of potential bias was related to confounding, which was rated as moderate across all studies due to the observational nature of the designs and the limited control of potential confounding variables. Bias in the classification of interventions, measurement of outcomes, and selection of the reported results was considered low risk in all studies. Regarding the selection of participants, most studies were judged as low risk, except for one study that presented moderate risk due to a convenience sample. In addition, missing data were judged as low risk in some studies and moderate risk in others due to incomplete reporting of follow-up data.

### 3.4. Results of Syntheses

Due to differences in follow-up periods and outcome definitions across studies, results are presented descriptively (Table 2).

#### 3.4.1. Linear Soft-Tissue Changes

Linear soft-tissue outcomes included pontic height (PH), mesial abutment height (MA), distal abutment height (DA), and horizontal ridge width measured at different levels below the ridge crest.

#### 3.4.2. Pontic Height

Overall, pontic height changes were limited over time. At one year, pontic crown height decreased by −1.24 mm in connective tissue graft (CTG) sites and −0.22 mm in control sites [3]. At five years, PH changes of −0.34 ± 0.5 mm in CTG sites and −0.35 ± 0.2 mm in control sites were reported [13]. At ten years, PH changes were −0.33 mm in CTG sites and −0.17 mm in controls [15]. At fifteen years, PH changes were −0.47 mm in CTG sites and 0.00 mm in control sites [12].

#### 3.4.3. Abutment Height

Changes in abutment height were reported at the fifteen-year follow-up. Mesial abutment height changes were −0.72 mm in CTG sites and −0.51 mm in control sites, while distal abutment height changes were −0.34 mm and −0.82 mm, respectively [12].

#### 3.4.4. Ridge Width

Horizontal ridge-width measurements showed variable changes over time. At one year, ridge width increased by +2.23 mm in CTG sites, whereas a slight reduction of −0.12 mm was observed in control sites [3]. At five years, reductions in CTG sites were −0.31 ± 0.1 mm (RW1), −0.37 ± 0.2 mm (RW3), and −0.42 ± 0.2 mm (RW5) [13]. At ten years, ridge-width reduction reached −0.62 mm in CTG sites and −0.20 mm in control sites at the 1 mm subcrestal level [15]. At fifteen years, reductions of −1.05 mm in CTG sites and −0.38 mm in control sites were reported [12].

#### 3.4.5. Volumetric and Profilometric Outcomes

Volumetric and profilometric analyses were performed using three-dimensional STL superimposition. At six months, volumetric changes of +1.41 ± 0.08 mm^3^ in CTG sites and +1.40 ± 0.08 mm^3^ in control sites were reported [14]. An additional study evaluating post-extraction ridge preservation reported volumetric reductions ranging from −1.78 to −1.15 mm^3^ at 6 months [20]. At five years, volumetric reductions of −5.31 ± 1.1 mm^3^ in CTG sites and −4.32 ± 1.7 mm^3^ in controls were observed [13].

Profilometric analysis demonstrated dimensional contour changes over time. At one year, contour changes of +0.61 mm in CTG sites and −0.25 mm in control sites were reported [3]. At five years, mean distance (MD) changes were −0.19 ± 0.5 mm in CTG sites and −0.16 ± 0.3 mm in control sites [13]. At ten years, MD changes of −0.64 mm in CTG sites and −0.22 mm in controls were reported [15], while another long-term evaluation reported profilometric changes of 0.00 ± 0.37 mm in CTG sites and −0.03 ± 0.10 mm in control sites at the same follow-up period [16]. At fifteen years, MD changes of −0.68 mm in CTG sites and −0.33 mm in control sites were observed [12].

Additionally, soft-tissue shrinkage of 6.4% in CTG sites and 47% in control sites from baseline to six months was reported [14].

### 3.5. Reporting Biases

Across the included studies, no clear evidence of reporting bias due to missing results was identified. Most studies reported the outcomes described in their Methods Sections, and no selective outcome reporting was apparent. However, only a limited number of studies had publicly available protocols or trial registrations, which restricts the ability to fully exclude selective reporting. Given the small number of included studies and the consistency of reported outcomes across studies, the overall risk of bias due to missing results was judged to be low to unclear.

### 3.6. Certainty of Evidence

The certainty of the evidence for the outcome dimensional changes in soft tissue was assessed using the GRADE approach. Two randomized controlled trials [14,20] and three non-randomized studies [3,12,16] contributed to this outcome. An important consideration was the presence of multiple publications derived from the same study cohort at different follow-up periods [12,13,15]. To avoid duplication and overestimation of effects, only the most recent data were included in the synthesis [12].

For the randomized trials [14,20], the certainty of evidence was downgraded due to concerns related to risk of bias, inconsistency, and imprecision. Risk of bias was judged as serious because both studies presented concerns in the domain of missing outcome data. Inconsistency was also rated as serious due to important clinical and methodological heterogeneity across studies, including variations in surgical techniques, follow-up periods, and outcome measurement methods. Although the direction of effect was generally consistent, these differences limited comparability. Imprecision was considered serious due to the small sample sizes and the limited number of included studies. Additionally, the absence of meta-analysis prevented the calculation of pooled estimates and confidence intervals, further limiting the robustness of the findings. No serious concerns were identified regarding indirectness.

For the non-randomized studies [3,12,16], the certainty of evidence was likewise rated as very low. The evidence was downgraded for risk of bias, primarily due to methodological limitations inherent to non-randomized designs, including potential confounding and concerns related to missing outcome data, as assessed using the ROBINS-I tool. Similar to the randomized studies, inconsistency and imprecision were rated as serious due to heterogeneity across studies and small sample sizes. Indirectness was not considered a concern, as the included studies directly addressed the review question.

Overall, the combination of methodological limitations, heterogeneity, and imprecision substantially reduced confidence in the estimated effects, leading to a very low certainty of evidence, indicating that the true effect is likely to be substantially different from the observed estimates. More information can be found in Table 3.

## 4. Discussion

This systematic review examined volumetric and linear soft-tissue changes at pontic sites treated with CTG or left to heal without grafting. Overall, the available evidence suggests that CTG may provide early improvements in ridge contour and soft-tissue dimensions; however, the long-term advantage over nongrafted healing appears less certain. Because the certainty of evidence was very low, these findings should be interpreted cautiously and should not be considered definitive evidence of superiority.

Based on the included studies, early dimensional reductions after CTG most likely reflect physiological tissue remodeling rather than graft failure. This early phase has been associated with contraction, revascularization, fibroblast reorientation, and collagen maturation, as described in the included studies [13,15,16]. These processes may explain why most dimensional changes occur during the initial healing period and then tend to stabilize over time, which is consistent with remodeling patterns reported in periodontal and mucogingival CTG applications [14,20].

Vertical outcomes, including pontic height (PH) and abutment heights (MA, DA), generally showed limited changes across mid- and long-term follow-up periods. Strauss et al. (2022) reported early improvements in contour during the first year after CTG [3]. Longer-term evaluations indicated relatively small alterations in pontic and abutment height measurements, including at the 15-year follow-up [12,15]. Nevertheless, the small number of studies and methodological heterogeneity limit the strength of this interpretation.

For horizontal ridge dimensions, CTG was associated with greater early gains in ridge width during the first year [3]. However, longer follow-up studies showed that differences between grafted and nongrafted sites tended to diminish over time, with reported changes often remaining within a range that may be difficult to perceive clinically [13,15,16]. These findings suggest that long-term soft-tissue contour stability may depend not only on grafting but also on prosthetic design, pontic form, tissue support, and maintenance conditions. The broader prosthetic literature indicates that ovate and modified ridge-lap pontic designs may help distribute pressure, support papilla architecture, and facilitate hygiene [11,12,15,16]. Therefore, surgical augmentation and prosthetic planning should be considered interdependent rather than separate determinants of pontic-site stability.

Volumetric analyses generally followed the same pattern as linear measurements. CTG sites tended to show early volume gain or improved contour, whereas between-group differences appeared to diminish with longer follow-up [13,14,15,16,20]. However, because measurement protocols, follow-up periods, and study designs varied substantially, these findings should be interpreted as descriptive trends rather than conclusive comparative evidence. Evidence outside the present synthesis has explored alternative materials and techniques, including cross-linked collagen matrices, with some studies reporting comparable short-term outcomes and differences in remodeling behavior [22,23]. These findings provide useful context but should not be interpreted as direct evidence within the present review.

Although CTG is widely regarded as a reference approach for soft-tissue volume enhancement, particularly for its biological integration and established clinical use, evidence from implant-based studies should be extrapolated to pontic sites cautiously [12,15,24,25]. Collagen matrices may be considered when autogenous graft harvesting is limited by patient morbidity, anatomical factors, or surgical preference, but their long-term volumetric stability at pontic sites remains less well established [22,24,25]. Within the limits of the available evidence, CTG may be most relevant for esthetically demanding or ridge-deficient pontic sites rather than as a routine intervention for all cases [4,5,11,12,13,15,16,20]. In addition to increasing tissue volume, CTG may support the mucosal zenith, emergence profile, and papillary architecture [11,12]. However, future randomized clinical trials should include patient-reported esthetic satisfaction, morbidity, comfort, and maintenance burden to clarify whether measurable dimensional changes translate into clinically meaningful benefit.

Measurement variability remains an important issue. Studies using analog impressions and indirect digitization may be affected by distortion of impression material, stone expansion, and conversion-related inaccuracies. In contrast, digital intraoral scanning with standardized region-of-interest (ROI) superimposition may improve reproducibility and reduce operator-dependent variation. External validation studies of optical systems have reported measurement deviations mostly within the 80–120 μm range, suggesting clinically acceptable precision for longitudinal volumetric assessment [13,15,26,27]. Future studies should standardize ROI definitions, follow-up intervals, outcome reporting, and the combined use of mean distance and volumetric change to improve comparability across studies.

The evidence base was small, and most included studies were non-randomized, increasing the risk of confounding and limiting causal interpretation. In addition, several studies were conducted by a limited number of research groups, with a predominance of Zurich-based cohorts. This concentration of evidence may restrict generalizability because similar protocols, operators, measurement workflows, and patient populations may have influenced the reported outcomes. Reporting bias also cannot be excluded. The small number of included studies precluded formal assessment using funnel plots or statistical tests. Furthermore, the lack of patient-centered and histological outcomes limits the interpretation of soft-tissue quality beyond dimensional change. Finally, because clinical and methodological heterogeneity prevented meta-analysis, the findings remain descriptive and should be interpreted with caution.

In summary, CTG appears to provide early soft-tissue augmentation at pontic sites and may help maintain contour over time. However, its long-term advantage over non-grafted healing remains uncertain and seems to diminish after tissue maturation. Given the very low certainty of evidence, CTG should be considered selectively, particularly in esthetic or ridge-deficient sites, and the variations in results should be communicated to the patients. Further standardized volumetric measurement protocols should be developed.

## 5. Conclusions

Connective tissue grafting (CTG) at pontic sites appears to provide early soft-tissue augmentation and may support contour stability, but long-term differences compared with non-grafted healing tend to diminish over time, and their clinical relevance remains uncertain.

Given the very low certainty of evidence, these findings should be interpreted with caution, and CTG should be considered selectively, particularly in esthetic or ridge-deficient cases.

## Figures and Tables

**Table 1:** Characteristics of Included Studies. Summary of the six included studies evaluating soft-tissue dimensional and volumetric outcomes after CTG at pontic sites.

Author (Year) Study Design	Impression/Model	Digital Analysis	ROI Definition	Sample (Test/Control)	Follow-Up	Intervention	Comparison	Primary Outcomes	Main Findings
Akcalı et al., 2015 RCT [14]	Silicone impression (GC Exaflex); dental stone model	Optical scanner (Imetric 3D); Swissmeda/SMOP	Mesial/distal papilla–mucogingival line–alveolar crest	20 (10/10)	6 mo	Modified VIP-CTG	Free SCTG	VC, MD	Minor gain; shrinkage lower in CTG (6.4% vs. 47%).
Schneider et al., 2014 RCT [20]	Silicone + CBCT	Optical scanner; Swissmeda/SMOP	Buccal area between papillae and mucogingival line	20 (10/10)	6 mo	DBBM-C/PG	β-TCP, DBBM-C/CM, no graft	MD	Slightly less volume loss in CTG vs. controls; ns.
* Sanz-Martín et al., 2016 Controlled Clinical Trial [13]	Alginate impressions	Optical scanner; Swissmeda/SMOP	Mucosal margin to apical 5–6 mm ROI	24 (12/12)	5 y	Subepithelial CTG	No graft	PH, MA, DA, RW, VC, MD	Minor reduction; no significant difference.
* Bienz et al., 2017 Controlled Clinical Trial [15]	Alginate impressions	Optical scanner; Swissmeda/SMOP	Pontic site ROI from mucosal margin to 5–6 mm apical	24 (12/12)	10 y	Subepithelial CTG	No graft	PH, MA, DA, RW, MD	Gradual reduction; no significant difference.
* Bienz et al., 2025 Controlled clinical study (15-year follow-up of previous RCT cohort) [12]	Alginate impressions	Optical scanner; Swissmeda/SMOP	ROI was defined with 1 mm dis- tance from the mucosal margin on the baseline STL. Mucosal margin at 5–6 mm apical	14 patients (8/6)	15 y	Subepithelial CTG	No graft	PH, RW, MD	Slightly greater reduction in augmented sites but no statistically significant intergroup differences.
Naenni et al., 2020 Retrospective [16]	A-silicone (President)	Optical scanner; Swissmeda/SMOP	Trapezoid ROI below gingival margin	15 (6/9)	10 y	Subepithelial CTG	RBFDP without graft	PH, RW	Minimal change; not significant.
Strauss et al., 2022 Cohort [3]	Digital + conventional impressions	3Shape + Imetric 3D; Swissmeda/SMOP	Buccal pontic ROI	24 (6/18)	1 y	Subepithelial CTG	No graft	PH, RW, MD	Short-term gain; ns.

* Studies marked with an asterisk (*) are based on the same clinical cohort and report outcomes at different follow-up time points.

**Table 2:** Summary of Linear, Volumetric, and Profilometric Soft-Tissue Changes at Pontic Sites.

Outcome	Follow-Up	CTG (Test)	Control	*p*-Value	Study
Pontic height (PH)	1 year	−1.24	−0.22	0.022	[3]
	5 years	−0.34 ± 0.5 mm	−0.35 ± 0.2 mm	>0.05	[13]
	10 years	−0.33 mm	−0.17 mm	>0.05	[15]
	15 years	−0.47 mm	0.00 mm	0.079	[12]
Mesial abutment height (MA)	15 years	−0.72 mm	−0.51 mm	NR	[12]
Distal abutment height (DA)	15 years	−0.34 mm	−0.82 mm	NR	[12]
Ridge width (RW1)	5 years	−0.31 ± 0.1 mm	−0.35 ± 0.2 mm	>0.05	[13]
Ridge width (RW3)	5 years	−0.37 ± 0.2 mm	−0.36 ± 0.2 mm	>0.05	[13]
Ridge width (RW5)	5 years	−0.42 ± 0.2 mm	−0.41 ± 0.2 mm	>0.05	[13]
Ridge width (1 mm subcrestal)	10 years	−0.62 mm	−0.20 mm	>0.05	[15]
Ridge width (overall)	1 year	+2.23 mm	−0.12 mm	0.032	[3]
	15 years	−1.05 mm	−0.38 mm	0.138	[12]
Volumetric change	6 months	−1.15 to −1.69 mm^3^	−1.78 mm^3^	>0.05	[20]
	6 months	+1.41 ± 0.08 mm^3^	+1.40 ± 0.08 mm^3^	0.93	[14]
	5 years	−5.31 ± 1.1 mm^3^	−4.32 ± 1.7 mm^3^	>0.05	[13]
Mean distance (MD)	1 year	+0.61 mm	−0.25 mm	0.038	[3]
	5 years	−0.19 ± 0.5 mm	−0.16 ± 0.3 mm	>0.05	[13]
	10 years	−0.64 mm	−0.22 mm	>0.05	[15]
	15 years	−0.68 mm	−0.33 mm	0.208	[12]
Soft-tissue shrinkage	6 months	6.4%	47%	>0.05	[14]

Abbreviations: CTG: connective tissue graft, NR: Not reported.

**Table 3:** Certainty of the evidence according to the GRADE approach for the outcome: dimensional changes in soft tissue.

Certainty Assessment							Certainty
№ of Studies	Study Design	Risk of Bias	Inconsistency	Indirectness	Imprecision	Other Considerations	
2	randomised trials	serious ^a^	serious ^c^	not serious ^d^	serious ^e^	none	⨁◯◯◯ Very low
3	non-randomised studies	serious ^b^	serious ^c^	not serious ^d^	serious ^e^	Multiple publications from the same study cohort at different follow-up periods were identified. To avoid duplication, only the most recent data were considered.	⨁◯◯◯ Very low

Explanations: ^a^. Both studies presented some concerns regarding the domain “Bias due to missing outcome data” in the risk of bias assessment. ^b^. Downgraded one levels due to important methodological limitations in the non-randomized studies, including potential confounding and concerns related to missing outcome data, as identified through the ROBINS-I assessment. ^c^. Downgraded one level in both study design due to important clinical and methodological heterogeneity across studies, including differences in surgical techniques, follow-up periods, and outcome measurement methods. Although the direction of effect was generally consistent, the variability in study characteristics limited comparability and reduced confidence in the consistency of the findings. ^d^. The included studies directly addressed the review question in terms of population, intervention, and outcomes. The outcomes assessed (e.g., dimensional changes in soft tissue) are clinically relevant and directly reflect the objective of the review, with no important concerns regarding indirectness. ^e^. Downgraded one level due to imprecision arising from small sample sizes and a limited number of studies, which reduce the robustness of the evidence. Furthermore, the absence of meta-analysis precluded the calculation of pooled estimates and confidence intervals, limiting the ability to assess the precision and stability of the effect estimates. As a result, the certainty of the evidence was reduced.

## Data Availability

Datasets used and/or analyzed during this study are available from the authors upon reasonable request.

## Supplementary Materials

The following supporting information can be downloaded at: https://www.mdpi.com/article/10.3390/jcm15103842/s1, **Supplementary Material S1.** PRISMA 2020 checklist indicating where each reporting item is addressed in the manuscript. **Supplementary Material S2.** Database Search strategies. **Supplementary Material S3.** Studies excluded after full-text assessment, along with the reasons for exclusion.
